# Chromosomal instability and acquired drug resistance in multiple myeloma

**DOI:** 10.18632/oncotarget.20829

**Published:** 2017-09-11

**Authors:** Wang Wang, Yi Zhang, Ruini Chen, Zhidan Tian, Yongpin Zhai, Siegfried Janz, Chunyan Gu, Ye Yang

**Affiliations:** ^1^ The Third Affiliated Hospital, Nanjing University of Chinese Medicine, Nanjing, 210023, China; ^2^ School of Medicine and Life Sciences, Nanjing University of Chinese Medicine, Nanjing, 210023, China; ^3^ Department of Burns and Plastic Surgery, Affiliated Hospital of Nantong University, Nantong, 226001, China; ^4^ Department of Pathology, Nanjing First Hospital, Nanjing, 210006, China; ^5^ Department of Hematology, Jinling Hospital, School of Medicine, Nanjing University, Nanjing, 210002, China; ^6^ Department of Pathology, The University of Iowa Roy J. and Lucille A. Carver College of Medicine, Iowa City, 52242, USA

**Keywords:** chromosomal instability, proliferation, drug resistance, multiple myeloma

## Abstract

Chromosomal instability (CIN) is an important hallmark of human cancer. CIN not only contributes to all stages of tumor development (initiation, promotion and progression) but also drives, in large measure, the acquisition of drug resistance by cancer cells. Although CIN is a cornerstone of the complex mutational architecture that underlies neoplastic cell development and tumor heterogeneity and has been tightly associated with treatment responses and survival of cancer patients, it may be one of the least understood features of the malignant phenotype in terms of genetic pathways and molecular mechanisms. Here we review new insights into the type of CIN seen in multiple myeloma (MM), a blood cancer of terminally differentiated, immunoglobulin-producing B-lymphocytes called plasma cells that remains incurable in the great majority of cases. We will consider bona fide myeloma CIN genes, methods for measuring CIN in myeloma cells, and novel approaches to CIN-targeted treatments of patients with myeloma. The new findings generate optimism that enhanced understanding of CIN will lead to the design and testing of new therapeutic strategies to overcome drug resistance in MM in the not-so-distant future.

## INTRODUCTION

Chromosomal instability (CIN) refers to a characteristic property of cancer cells, in which chromosomes are not as stable as they are in normal cells. It is common in cancer cells to find an increased rate of chromosome mal-segregation during mitosis [1]. An error of this sort may occur in 1–5 mitoses in cancer cells, but less than once in 100 mitoses in normal cells [2]. CIN is a type of genomic instability in which either whole chromosomes or parts of chromosomes are duplicated or deleted [3, 4] or the number of intact chromosomes is changed (aneuploidy). CIN always leads to aneuploidy; however, aneuploidy may not develop to CIN [5, 6].

CIN is divided into numerical chromosomal instability (n-CIN) and structural chromosomal instability (s-CIN) [7]. Numerical CIN is a gain or loss of whole chromosomes. Structural CIN leads to the duplication or deletion of chromosome fragments [8]. CIN is tightly associated with both tumorigenesis and outcome/prognosis of cancer. There is a remarkable correlation between n-CIN and s-CIN [5]. With regard to ploidy status of cells, CIN can occur in polyploid cells and diploid cells. Based on different phenotypes, CIN-high and CIN-low cells can be distinguished [9]. Furthermore, type I CIN can be distinguished from type II CIN in accordance with the underlying molecular mechanism [10].

Multiple myeloma (MM), also known as plasma cell myeloma, is the second most common hematologic malignancy. MM is treatable but not curable, with the latter being caused in large measure by the development of resistance to myeloma drugs due to CIN [11–15]. Hence, to achieve a cure for myeloma, it is crucial to increase our understanding of the mechanism by which CIN leads to drug resistance.

Proper chromosome segregation is an important guarantor of chromosome stability [16–18], as it ensures that one pair of sister chromatids, with it kinetochores properly attached to the microtubules from two opposing spindle poles, will be equally allocated to the daughter cells. Erroneous kinetochore–microtubule attachments may result in CIN despite the availability of a spindle assembly checkpoint (SAC) to avoid this from happening [19]. Merotelic attachment, one type of erroneous attachments, is a major cause of CIN [20], for its contribution to lagging chromosomes, which may eventually result in both n-CIN and s-CIN. Centrosome amplification [21], spindle assembly [22] and chromosome alignment defect, weakened activity of Aurora kinases and microtubule stabilization may all lead to Merotelic attachment [7]. In addition, defects in SAC [23], sister chromatid condensation or cohesion issues [24] and replication stress [25] may all result in CIN. Although it is difficult to integrate these mechanisms into one general mechanism, in the following we will attempt to outline the mechanism by which CIN impacts cell proliferation and drug resistance. Furthermore, we will discuss biological and molecular features of CIN in MM.

### Common regulatory factors of CIN in cell proliferation and drug resistance

**Figure 1:**
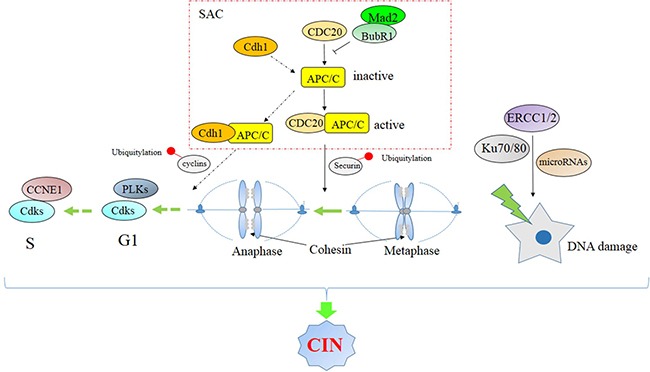
Signaling pathway associated with chromosomal instability

To describe the impact of CIN on cell proliferation and drug resistance in MM, we present recent research results on CIN in MM and consider the role of critical CIN factors in MMC proliferation and drug resistance (Table 1 and Figure 1). The spindle assembly checkpoint (SAC) is a major cell-cycle regulatory pathway that monitors the accuracy of chromosome segregation during mitosis. It ensures that sister chromatids will not separate until all chromosomes are attached to spindle microtubules. Compromised SAC function followed by CIN is the main cause of chromosomal mis-segregation. Mouse models exhibiting reduced expression of SAC genes show CIN and high rates of spontaneous tumor formation in most cases, supporting the conclusion that CIN promotes oncogenesis [26]. In addition, DNA damage repair factors, cyclin dependent kinase, microRNAs and cancer microenvironment factors are involved.

**Table 1:** Influence factors of CIN contributing to drug resistance in MM

CATEGORY	GENE SYMBOL	GENE/PROTEIN FUNCTION	REFs
SAC related gene	*MAD2*	Essential spindle checkpoint protein	[23]
	*BUB1*	Expression in myeloma cells is highly correlated to CDC20 and CCNB1/2 expression, and leads to increased myeloma proliferation.	[85]
	*MPS1*	Monopolar spindle 1 is a kinase that has key functions in activating SAC, which effects the proper distribution of chromosomes to daughter cells.	[86]
	*Aurora B*	Aurora B kinase activity results in the attachment of the mitotic spindle to the centromere. Gene expression in multiple myeloma is associated with genetic instability and increased cell proliferation.	[17]
	*NEK2*	NIMA-related kinase 2 induces drug resistance in myeloma by virtue of activating drug efflux pumps. Gene overexpression results in CIN in many types of cancer.	[12, 28]
	*PLKs*	Play important roles in cell cycle progression, checkpoint control, and mitosis.	[87]
Cyclins	*CCND1*	Upregulation of cyclin D1 in cancer cells is associated with genomic instability and resistance to DNA-damaging drugs.	[34]
	*CCNE1*	Overexpression results in CIN and reduced sensitivity of myeloma cells to the CDK inhibitor, seliciclib.	[35]
	*CDK1*	Cyclin-dependent kinase 1, an important cell cycle regulator, may be deregulated in multiple myeloma.	[88]
DNA repair related gene	*XRCC5/6*	Encode the nuclear proteins, Ku70 and Ku80, which bind to DNA double-strand breaks (DSBs) and are important for DSB repair, telomere maintenance, and regulation of programmed cell death (apoptosis).	[89, 90]
	*ERCC1/2*	ERCC1 protein plays a key role in nucleotide excision repair. ERCC1 dimerizes with xeroderma pigmentosum complementation group F, and this complex is required for the excision of the damaged DNA.	[91, 92]
	PARP1/2	Required for DNA single-strand break repair. Inhibition of gene function causes DNA replication fork collapse and DNA DSBs.	[93]
	MMSET/WHSC1	Histone methyl transferase overexpressed in t(4;14)^+^ MM. Inhibition of MMSET results in enhanced efficacy of chemotherapy, reduced myeloma growth, and extended survival of patients with myeloma.	[94, 95]
APC/C related gene	*Cdc20*	E3 ubiquitin ligase involved in cell cycle regulation	[23, 85]
	*Cdh1*	Cell-cycle regulated activator of the APC/C, which suppresses the re-accumulation of mitotic cyclins and stabilizes the G1 phase of the cell cycle.	[96, 97]
microRNAs	miR-137	Epigenetically silenced in MM. Over expression may overcome CIN and drug resistance of myeloma cells by virtue of impacting DNA damage repair pathways.	[37]
	miR-433	Aberrant expression may adversely affect intracellular signaling in osteoclasts and, thereby, promote chemoresistance and cellular senescence in myeloma.	[98]
Phospho-inositide Pathway Gene	PI3K/AKT, PTEN	PTEN is a tumor suppressor. Its loss leads to activation of the oncogenic PI3K/AKT/mTOR pathway which promotes tumor development and progression.	[99]
ALDH1 family	ALDH1A1	Over expression in myeloma cells may lead to increased mRNA and protein levels of NEK2, to elevated clonogenicity of myeloma and tumor formation in mice, and to resistance to myeloma drugs *in vitro* and *in vivo.*	[30]

### SAC-related factors

SAC-related factors include MAD2, BUB1, MPS1, Aurora B, NEK2 and PLKs. Mitotic arrest deficient 2 (MAD2) is an essential spindle checkpoint protein that restrains progression through the metaphase-to-anaphase transition. It binds either CDC20 or MAD1 to regulate the cell cycle. BUB1B expression is highly correlated to CDC20 and CCNB1/2 expression in MMCs and leads to elevated MM cell proliferation [27].

NIMA-related kinase 2 (NEK2), a novel CIN gene of great interest for myeloma research, was first reported by the Schultz Lab [28]. NEK2 is closely linked to drug resistance and poor prognosis in multiple cancers. Zhan FH et al. [12] reported that overexpression of NEK2 in cancer cells results in enhanced CIN, vigorous cell proliferation, and significant levels of drug resistance. In contrast, knocking down NEK2 by shRNA reversed these phenotypes *in vitro* and in a xenograft myeloma mouse model *in vivo*. Clearly, NEK2 is a promising therapeutic target in MM [12]. Aldehyde dehydrogenase 1 (ALDH1) is also involved in NEK2-dependent CIN in myeloma. ALDH1 is a member of the ALDH family of proteins, which contributes to the survival of human multiple myeloma stem cells (MMSCs). It leads to increased NEK2 expression and plays an important role in drug resistance. ALDH1 expressing or ALDH1^+^ cells were interrogated to evaluate ALDH1 function in myeloma growth [29]. ALDH1A1, one of several member of the ALDH1 family, can be readily detected in myeloma cells. Overexpression of ALDH1A1 leads to increased clonogenicity of myeoma cells, tumor formation in mice, and resistance to chemotherapy drugs *in vitro* and *in vivo*. Yang et al. demonstrated that the ALDH1A1-RXRα-NEK2 signaling pathway may play a key role in acquired drug resistance and disease relapse in myeloma, suggesting that specific inhibitors of ALDH1A1 are worthy for development of new myeloma treatments [30].

Polo-like kinases (PLKs) are crucial for cell cycle progression, checkpoint control, and mitosis. In cells undergoing mitosis, PLKs are associated with the centrosome, kinetochores and the central spindle apparatus. Increased PLK expression in tumors is tightly correlated with chromosomal instability, centrosome amplification, and DNA aneuploidy [31]. The high mitotic index and chromosomal instability of advanced cancers suggest that PLK inhibitors may be an attractive therapeutic option for multiple myeloma.

### Ku proteins

To date, it is difficult to pin the exact mechanism by which CIN increases proliferation and drug resistance in MM. However, recent studies are beginning to view this situation in the context of DNA damage and epigenetic changes. Gullo et al. [32] reported that Ku – a heterodimeric protein that consists of two subunits, binds to DNA double-strand break ends, and modulate DNA repair & telomere maintenance pathways – is crucial for maintaining chromosomal stability. Overexpression of Ku proteins promotes cell proliferation and resistance to apoptosis. Conversely, deficiency or low-level expression of Ku leads to genomic instability and tumorigenesis. Thus, Ku may be an attractive therapeutic target in MM, and the isolation of a specific human monoclonal antibody to Ku86 may be an important step towards developing Ku-targeted immunotherapies [33].

### Cyclin E

Genomic instability was thought to be influenced by elevated cyclin E (CCNE), one regulator of cyclin dependent kinase (CDK) in Spruck C H, et al's work [34]. T Benyehoshua, L J, et al's his study examined the functions of CCNE in MM and revealed that CCNE1 expression was heterogeneous in various MMCs. CDK may induce drug resistance by enhancing the ability of adhesion of MMCs to fibronectin (FN). Seliciclib, a selective CDK-inhibitor, inhibits adhesion of MMCs to FN, which induces apoptosis through the pathway of MCL1 and p27. Overexpression of CCNE1 leads to drug resistance, whereas CCNE1 silenced by siRNA improves the sensitivity to seliciclib in MMCs. It is concluded that seliciclib may act as essential component of modern anti MM drug combination therapy [35].

### Deregulated microRNAs

Aberrant microRNAs or “miRs” are endogenous, single-stranded, non-coding RNAs 19–25 nucleotides in length, which regulate gene expression by targeting the 3′-untranslated region (3′-UTR) of mRNAs [36]. Qin *et al*. showed that miR-137 is significantly decreased in MM and associated with poor outcome. These investigators elucidated the epigenetic regulation of miR-137 and its association with progression-free survival of MM patients. Furthermore, overexpression of miR-137 in myeloma cells, designated miR-137^OE^, enhanced their sensitivity to bortezomib and eprirubicin *in vitro*. Moreover, certain high-risk genetic abnormalities in MM, such as deletion of chromosome 1p22.2, 14q or 17p13 and gain of chromosome 1p22.2, were detected in parental myeloma cells (NCI-H929 cells transfected with “empty” vector) treated with drugs, but not in miR-137^OE^ cells. Luciferase reporter assays demonstrated that miR-137 interacts with Aurora kinase A (AURKA). Ectopic expression of miR-137 strongly reduced the expression of both AURKA and p-ATM/Chk2, but resulted in upregulation of p53 and p21. Interestingly, overexpression of miR-137 in myeloma treated with bortezomib significantly inhibited tumor growth in a human-in-mouse xenograft model. Taken together, these studies revealed that miR-137 is epigenetically silenced in MM, and overexpression of miR-137 may reduce drug resistance and overcome chromosomal instability in MMCs via affecting the apoptosis and DNA damage pathways [37, 38].

The level of miR-19a may be useful to identify patients with high-risk MM, albeit additional research is warranted to improve the predictive value of this miR-19 in the different genetic subgroups of MM [39]. Two additional miRs, miR-195 [40] and miR-497 [41], were recently shown to be closely associated with checkpoint kinase; however, the significance of this finding for MM remains unclear at this juncture.

### PI3K/AKT pathway genes

APC/C-related and phosphoinositide pathway genes, such as *CDC20, CDH11*, *PTEN* and others, may also be involved in CIN in myeloma. In turn, this may impact myeloma cell proliferation, acquisition of drug resistance and other biological features of myeloma cells.

### Role of the tumor microenvironment (TME)

Chromosome instability and drug resistance can be caused not only by tumor cell-intrinsic mechanisms but also tumor-extrinsic pathways including tumor-stroma interactions. Indeed, stromal elements in the bone marrow TME, such as fibroblasts, osteoblasts, osteoclasts and immune cells, make important contributions to CIN and drug resistance in myeloma [42]. The myeloma TME contains a variety of MM stromal cells (MMSCs) which are involved in more ways than one in myeloma progression and therapy resistance. Myeloma cells home to the bone marrow TME, which provides a rich soil and safe haven for the neoplastic plasma cells. MMSCs are believed to promote genetic instability and drug resistance of myeloma by virtue of a complex mechanism that includes the provision of anti-apoptotic factors and cell adhesion-induced growth arrest.

A large body of evidence indicates that the physical interaction of myeloma cells and MMSCs (cell adherence) induces the non-malignant bystander cells to secrete cyto- and chemokines, such as IL-6, CD40, TRANCE and Ras, which collectively promote myeloma proliferation and survival [43, 44]. IL-6, a major growth and survival factor for normal and malignant plasma cells, activates the JAK-STAT and RAS pathways, which, in turn, further stimulate IL-6 production [45]. TRANCE, which is officially designated tumor necrosis factor ligand superfamily member 11 (TNFSF11), has been identified to induce osteoclast differentiation, and to control bone regeneration and remodeling. Increased osteoclastic activity results in the release of several cytokines, including IL-6, TGF-β and FGF, from the bone matrix. In turn, these cytokines promote myeloma proliferation. Elevated IL-6 signaling may lead to heightened expression of Bcl-xL [46], which in concert with active STAT-3 signaling may induce multidrug resistance in myeloma [47]. Overexpression of survival-enhancing Bcl-2 and Bcl-xL proteins makes it possible to accumulate mutations in myeloma cells and to tolerate aberrant chromosome segregation leading to CIN [48].

Changes in TME have also been shown to promote CIN cell lines other than myeloma [49]. For example, Huang *et al.* [50] found that stimulation with TGF-β1 induced many abnormal mitotic patterns, including lagging chromosomes and anaphase bridges, in NCM460 cells. Zheng *et al.* demonstrated that chromosome arm instability may result from telomere attrition in tumor cells. Interestingly, telomere length in carcinoma-associated fibroblasts (CAFs) is significantly associated with chromosomal instability and telomere length at 4q and 13q in lymphocytes, strongly suggesting that loss of genetic integrity in bystander cells promotes CIN in tumor cells 15q [51]. In sum, stromal elements in the bone marrow contribute to CIN and drug resistance in myeloma. However, the precise role the interaction of tumor cells with the TME plays in this process is poorly defined. Hence, additional research is warranted to shed light on the underlying molecular mechanisms, which may result in new therapeutic approaches for patients with MM.

### CIN may be a double-edged sword in myeloma development

There is ample evidence that CIN is a multifactorial phenotype that may be caused by a variety of molecular pathways also implicated in drug resistance of cancer cells and/or poor prognosis of patients with cancer. Clearly, additional work is warranted to better understand the association of CIN with these and other aspects of cancer. On this backdrop, it may be important to note that – although it is generally agreed that CIN promotes tumor progression in the great majority of circumstances – some results point to role of CIN as a double-edged sword for tumor development; that is, CIN may promote or suppress tumor initiation and progression depending on cellular context and magnitude of CIN [52]. A moderate amount of CIN may promote neoplasia via loss of chromosomal regions that contain tumor suppressor genes [53] and/or duplication/amplification of chromosomal regions that harbor oncogenes [54]. In contrast, large amounts of CIN may be lethal to cancer cells due to genomic catastrophe or chaos [55]. With respect to myeloma, too much CIN may exceed the capability of myeloma precursors to repair and recover from damage, while a tolerable level of CIN provides just the right mutational environment that supports malignant cell transformation [56]. The challenge for future myeloma research is to define the threshold at which the tumor-promoting role of CIN turns over to tumor inhibition. Enhancing CIN on purpose, by pharmacological means, may be of therapeutic benefit for patients with myeloma.

### Methods of measuring CIN

CIN-dependent changes of the genetic make-up of cell populations can be quantitatively determined, followed by validation using statistical methods. CIN may result in two types of changes: numerical and structural ones. Gain or loss of whole chromosomes (aneuploidy) or portions of whole chromosomes represent numerical changes, while structural change takes many forms that can be detected by a variety of methods including NCCAs [57]. Cytogenetic analytical techniques of CIN assessment include conventional and spectral karyotyping, fluorescence *in situ* hybridization (FISH), comparative genomic hybridization (CGH) and flow cytometry [57].

Swanton *et al.* [58] identified several genes that are overexpressed in tumors that exhibit CIN. They went on to show that these genes are involved in DNA repair, contribute to the survival of aneuploid cells, and can be repressed by treatment of cells with microtubule-stabilizing agents. They concluded that the expression level of these genes may be used as molecular signature of CIN. Julieta *et al.* [59] showed that RPA1 is overexpressed in MM, where it may link telomerase activity, telomere homeostasis and replication of chromosome ends with cell cycle progression. Weinhold *et al.* employed karyotype analysis to defined subgroups of multiple myeloma by virtue of specific chromosomal abnormalities [60]. Spectral karyotyping (SKY) and locus-specific FISH led to the identification of MM patients that exhibited focal amplifications of a certain receptor locus [61]. CGH is a method for detecting unbalanced DNA copy number variations (deletions, amplifications) that can be further validated using FISH. Array CGH, which permits increased throughput compared to conventional CGH, has been successfully used for “first-tier testing” in the diagnostic workup of MM [62]. Each method has advantages and disadvantages, and none of them is ideal for all applications. In general, one must use a battery of assays to evaluate CIN comprehensively and accurately in myeloma.

Lee *et al.* [63] developed a quantitative assay for measuring a particular type of CIN: chromosome missegregation. The assay makes use of a human artificial chromosome (HAC) that carries a constitutively expressed enhanced green fluorescence (EGFP) transgene. Cells that contain the HAC display green fluorescence, while cells that lack it do not. The rate at which HAC is lost, which has been shown to be mainly determined by CIN-dependent chromosome missegregation, can be readily measured with the help of flow cytometry. The impact of anticancer drugs that may lead to low levels of CIN that are difficult to detect using conventional methods was successfully evaluated in this manner way. The HAC-based assay has been recently refined by virtue of a shRNA-dependent genetic switch of EGFP expression [64] and a number of additional, nifty genetic modifications [65]. These permit one to accurately measure drug-dependent CIN in cancer cells, including myeloma, in which obvious mitotic defects are absent. HAC-harboring myeloma cells may lend themselves to detecting CIN below the threshold required for discernable morphological disruption.

### Myeloma drug-dependent CIN

Myeloma drugs include proteasome inhibitors, immunomodulatory drugs (IMiDs), glucocorticoids and conventional chemotherapeutics. Drug treatment is usually combined with autologous stem cell transplantation (ASCT) if the patient is eligible. Bortezomib, the first-in-class proteasome inhibitor, is widely used in myeloma therapy to inhibit cell cycle progression, myeloma growth and DNA damage repair, and to induce apoptosis in myeloma cells [66, 67]. IMiDs, such as thalidomide, lenalidomide and pomalidomide, are also incorporated in most therapeutic regimens of MM [68–70]. IMiDs modulate cytokine secretion, prevent MMCs from binding to BMSCs, induce MMCs apoptosis, and upregulate T cell and NK cell activity [71]. Glucocorticoids include dexamethasone and prednisone. Conventional chemotherapeutic drugs, such as doxorubicin, cyclophosphamide and melphalan, are also widely used for MM treatment, albeit mostly in combination with proteasome inhibitors and/or IMiDs [72].

**Figure 2:**
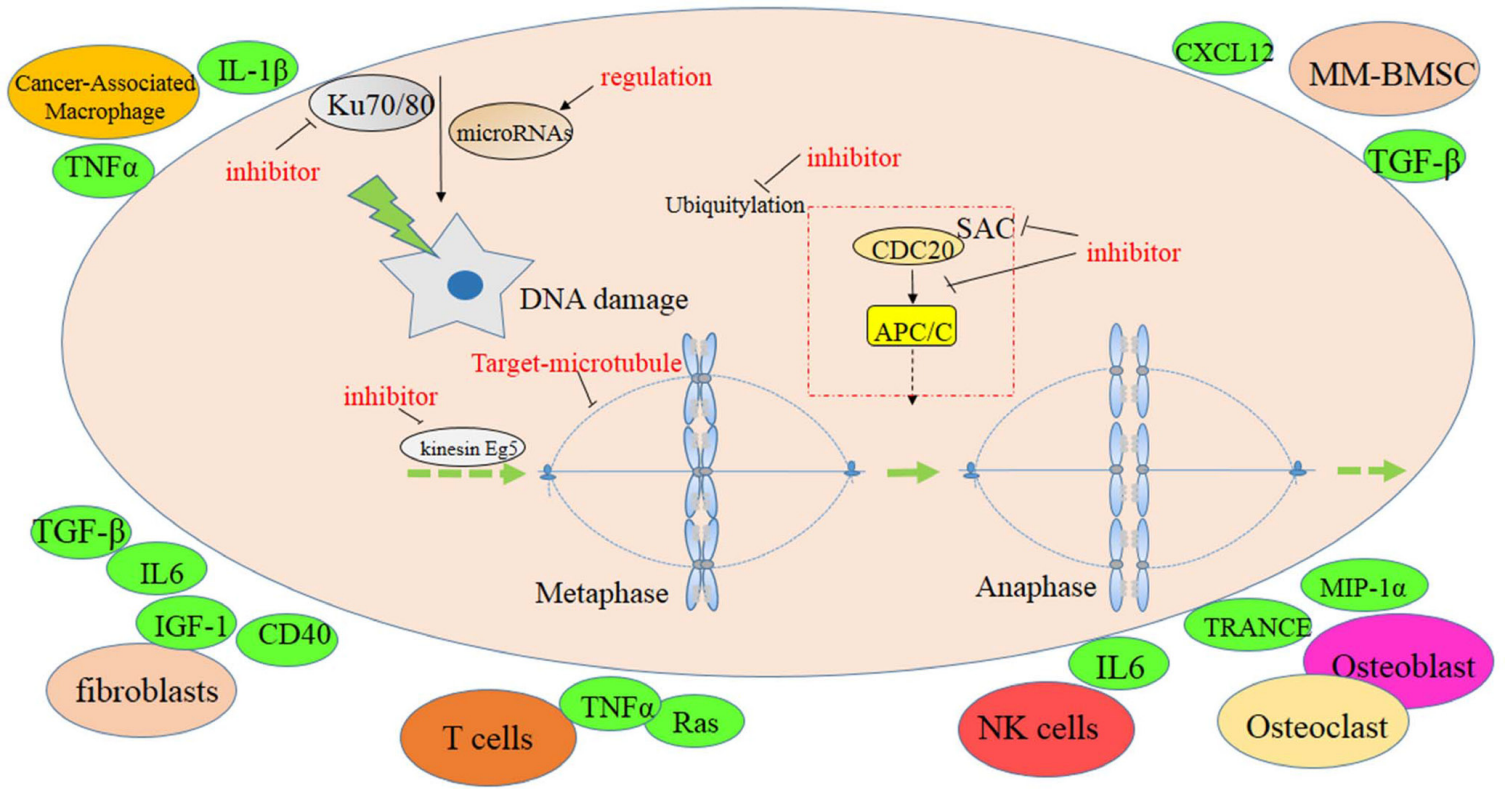
Potential targets to CIN in MM

CIN is closely associated with poor prognosis in myeloma. Molecular genetic tools, such as FISH analysis, indicate that 14q32 translocations [73], chromosome 13 deletion [74] and 17p13 deletion [75] in myeloma are correlated with poor prognosis. Drugs targeting CIN can improve poor prognosis and overcome drug resistance (Figure 2). As loss of mitotic fidelity is the prime cause of CIN, and SAC is the major pathway for enabling accurate chromosome segregation during mitosis, it may be worthwhile to evaluate anti-mitotic drugs, such as SAC inhibitors, for myeloma treatment. Microtubule-targeting agents like paclitaxel and vinca alkaloids may also be useful [76]. Inhibitors of mitotic kinesin Eg5 disturb the bipolar configuration of mitotic cells, which may result in monopolar spindle formation, prolong mitotic arrest, and cause apoptosis [77]. It has been reported that chemical inhibitors disturbing the interaction of CDC20 and APC/C block mitotic progress [78], suggesting that inhibitors of this sort might also be useful for MM. We believe that inhibitors of the following targets hold a great deal of promise for myeloma therapy: aurora kinase A [79], NEK2 [12], PLKs [80] and Mps1 kinase [81]. DNA-damaging agents and targeted DNA damage repair inhibitors such as cisplatin [82] and gemcitabine [83] may also be considered. In addition, drugs targeting the BM TME are expected to result in further improvement of myeloma therapy. For example, inhibitors of the CXCL12/CXCR4 interaction have therapeutic efficacy because they separate the neoplastic plasma cell from the protective BM milieu [84]. Seliciclib, a selective CDK inhibitor that blocks the adhesion of myeloma cells to FN, may further enrich the armamentarium of novel myeloma drugs [35].

## CONCLUSIONS

In summary, evidence indicates that CIN underlies acquired drug resistance in multiple myeloma and drives tumor heterogeneity in patients with myeloma. Targeting CIN upfront may be a viable approach to prevent genetic heterogeneity from occurring. Enhanced understanding of the pathophysiology of CIN is necessary to further improve myeloma treatment and, eventually, achieve a cure for the majority of patients with MM. New experimental model systems including transgenic mouse models of CIN-dependent human myeloma are required to improve our ability to measures CIN and target it for therapeutic purposes. A key area of future research is the mechanism by which CIN promotes drug resistance in MM.
